# Analysis of *unc-62* expression pattern in *C. elegans* embryonic AWC neurons

**DOI:** 10.17912/micropub.biology.000530

**Published:** 2022-02-18

**Authors:** Yi-Wen Hsieh, Chiou-Fen Chuang

**Affiliations:** 1 Department of Biological Sciences, University of Illinois at Chicago; 2 Graduate Program in Neuroscience, University of Illinois at Chicago

## Abstract

The *Caenorhabditis* *elegans* UNC-62 homothorax/Meis/TALE homeodomain protein functions sequentially to regulate general identity of the AWC olfactory neuron pair and the stochastic choice of asymmetric AWC subtypes during embryogenesis. Here we analyze the expression pattern of *unc-62* during AWC development using an integrated *unc-62::GFP* fosmid rescuing transgene. UNC-62::GFP was not detected in AWC neurons in early or late embryos. These results are consistent with previous single-cell RNA sequencing data and also suggest an undetectable level of *unc-62* expression and/or low stability of UNC-62 protein in AWC neurons during embryogenesis.

**Figure 1. Expression patterns of an integrated unc-62::GFP fosmid transgene in embryonic AWC neurons:**
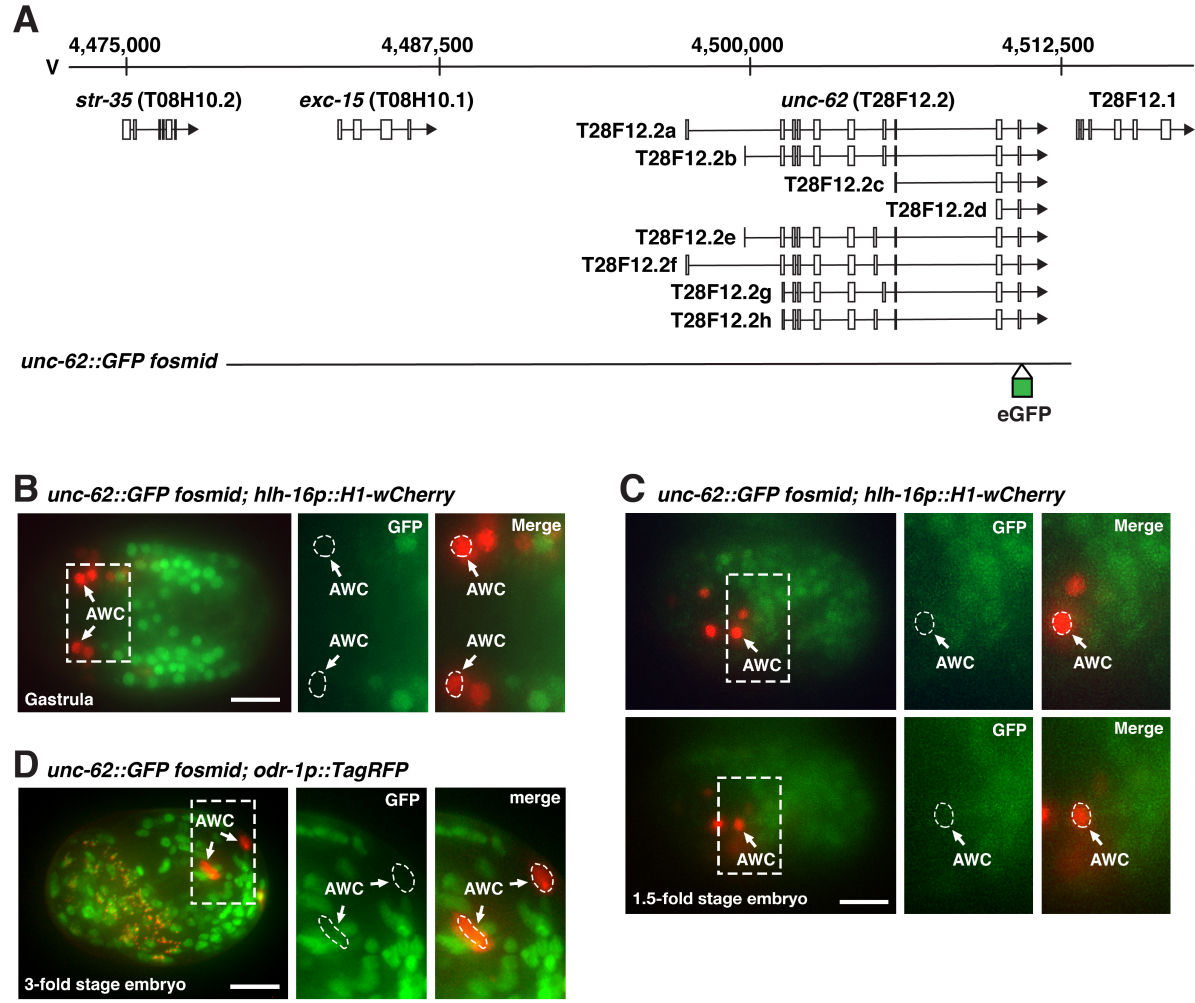
(**A**) Genomic structure and position of the *unc-62* locus and gene loci near *unc-62* in chromosome V. The genomic region of the *unc-62::GFP fosmid* clone is shown at the bottom. All UNC-62 protein isoforms are tagged with GFP at the C- terminus from the *unc-62::GFP* fosmid transgene (Van Nostrand *et al.*, 2013). (**B-D**) Representative images of UNC-62::GFP expression from an integrated *unc-62::GFP fosmid* transgene in a gastrula (B), a 1.5-fold stage embryo (C), and a 3-fold stage embryo (D). *hlh-16::H1-wCherry* and *odr-1p::TagRFP* expressed from integrated transgenes were used as early and late AWC markers, respectively. Insets in panels B-D are magnified by 2-fold. Scale bar, 10 um. Anterior to the left in B and C.

## Description

The UNC-62 homeodomain protein regulates AWC general identity and subsequently plays a cell autonomous role, determined by mosaic analysis, in AWC asymmetry during embryogenesis (Hsieh *et al.*, 2021). An integrated *unc-62::GFP* fosmid transgene, in which all UNC-62 protein isoforms are tagged with GFP at the C- terminus (Van Nostrand *et al.*, 2013) (Figure 1A), rescued *unc-62(lf)* mutant phenotypes of AWC general identity, determined by *odr-1p::DsRed* expression, and AWC asymmetry, determined by *str-2p::GFP* expression (Hsieh *et al.*, 2021). These results suggest that UNC-62::GFP fusion protein expressed from the *unc-62::GFP* fosmid transgene is functional for AWC development. It has been shown that this integrated *unc-62::GFP* fosmid transgene is expressed in sensory neurons, touch neurons, interneurons, ventral nerve cord motor neurons, and head motor neurons, but it is not expressed in AWC in late-stage larvae or young-stage adult worms using the multicolor transgene NeuroPAL (Reilly *et al.*, 2020).

The AWC neurons are born near the end of gastrulation; AWC asymmetry is established around the 1.5-fold and 3-fold embryonic stage (Sulston *et al.*, 1983; Chuang and Bargmann, 2005). To determine whether *unc-62* is expressed in AWC neurons at the embryonic stages of AWC development, the expression pattern of the integrated *unc-62::GFP* fosmid transgene (Van Nostrand *et al.*, 2013) (Figure 1A), was analyzed with integrated *hlh-16::H1-wCherry* or *odr-1p::TagRFP* transgene, early or late AWC marker, respectively. UNC-62::GFP was not detected in AWC neurons at the end of gastrulation, 1.5-fold, or 3-fold embryos (Figure 1B-D). Consistent with our results, single-cell RNA sequencing data revealed a very low expression level of *unc-62* in AWC during early embryogenesis as well as an undetectable level of *unc-62* in AWC in the later embryonic stage and second-larval stage (Cao *et al.*, 2017; Packer *et al.*, 2019). Together, these results suggest that *unc-62* may be expressed at an undetectable level and/or UNC-62 protein may have a very short half-life in embryonic AWC neurons.

## Reagents

**Table:** 

Strain	Genotype	Source
SD1871	*wgIs600* [*unc-62::GFP fosmid* (derived from *unc-62* fosmid clone WRM061dC01); *unc-119(+)*]	Van Nostrand *et al.*, 2013
RW10588	*unc-119(ed3); zuIs178 [his-72(1kb 5′ UTR)::his-72::SRPVAT::GFP::his-72 (1KB 3′ UTR) + 5.7 kb XbaI – HindIII unc-119(+)]; stIs10544 [hlh-16::H1-wCherry::let-858 3′ UTR]*	Murray *et al.*, 2012
IX5658	*wgIs600; stIs10544*	This study
IX3577	*wgIs600; vyIs56[odr-1p::TagRFP]* III (Cochella *et al.*, 2014)	This study
